# *Cannabis sativa*: origin and history, glandular trichome development, and cannabinoid biosynthesis

**DOI:** 10.1093/hr/uhad150

**Published:** 2023-07-26

**Authors:** Ziyan Xie, Yaolei Mi, Lingzhe Kong, Maolun Gao, Shanshan Chen, Weiqiang Chen, Xiangxiao Meng, Wei Sun, Shilin Chen, Zhichao Xu

**Affiliations:** Key Laboratory of Saline-alkali Vegetation Ecology Restoration (Northeast Forestry University), Ministry of Education, Harbin 150040, China; College of Life Science, Northeast Forestry University, Harbin 150040, China; Key Laboratory of Saline-alkali Vegetation Ecology Restoration (Northeast Forestry University), Ministry of Education, Harbin 150040, China; College of Life Science, Northeast Forestry University, Harbin 150040, China; Key Laboratory of Beijing for Identification and Safety Evaluation of Chinese Medicine, Institute of Chinese Materia Medica, China Academy of Chinese Medical Sciences, Beijing 100700, China; Key Laboratory of Saline-alkali Vegetation Ecology Restoration (Northeast Forestry University), Ministry of Education, Harbin 150040, China; College of Life Science, Northeast Forestry University, Harbin 150040, China; Key Laboratory of Saline-alkali Vegetation Ecology Restoration (Northeast Forestry University), Ministry of Education, Harbin 150040, China; College of Life Science, Northeast Forestry University, Harbin 150040, China; Key Laboratory of Beijing for Identification and Safety Evaluation of Chinese Medicine, Institute of Chinese Materia Medica, China Academy of Chinese Medical Sciences, Beijing 100700, China; Key Laboratory of Beijing for Identification and Safety Evaluation of Chinese Medicine, Institute of Chinese Materia Medica, China Academy of Chinese Medical Sciences, Beijing 100700, China; Key Laboratory of Beijing for Identification and Safety Evaluation of Chinese Medicine, Institute of Chinese Materia Medica, China Academy of Chinese Medical Sciences, Beijing 100700, China; College of Life Science, Northeast Forestry University, Harbin 150040, China; Key Laboratory of Beijing for Identification and Safety Evaluation of Chinese Medicine, Institute of Chinese Materia Medica, China Academy of Chinese Medical Sciences, Beijing 100700, China; College of Life Science, Northeast Forestry University, Harbin 150040, China; Institute of Herbgenomics, Chengdu University of Traditional Chinese Medicine, Chengdu 611137, China; Key Laboratory of Saline-alkali Vegetation Ecology Restoration (Northeast Forestry University), Ministry of Education, Harbin 150040, China; College of Life Science, Northeast Forestry University, Harbin 150040, China

## Abstract

Is *Cannabis* a boon or bane? *Cannabis sativa* has long been a versatile crop for fiber extraction (industrial hemp), traditional Chinese medicine (hemp seeds), and recreational drugs (marijuana). *Cannabis* faced global prohibition in the twentieth century because of the psychoactive properties of ∆^9^-tetrahydrocannabinol; however, recently, the perspective has changed with the recognition of additional therapeutic values, particularly the pharmacological potential of cannabidiol. A comprehensive understanding of the underlying mechanism of cannabinoid biosynthesis is necessary to cultivate and promote globally the medicinal application of *Cannabis* resources. Here, we comprehensively review the historical usage of *Cannabis*, biosynthesis of trichome-specific cannabinoids, regulatory network of trichome development, and synthetic biology of cannabinoids. This review provides valuable insights into the efficient biosynthesis and green production of cannabinoids, and the development and utilization of novel *Cannabis* varieties.

## Introduction

*Cannabis sativa* (Cannabaceae) is a diploid (2n = 20) dicotyledonous annual herbaceous plant and is distributed worldwide [1]. Historically, *Cannabis* has been grown as an important food, fiber, and medicinal crop. The whole *Cannabis* plant is used for various purposes. For example, its stems have been used for fiber production, which has been practiced for millennia. Its fruit, known as ‘huomaren’ in traditional Chinese medicine, functions as both medicine and food as it contains over 90 types of polyunsaturated fatty acids [2, 3]. Additionally, *Cannabis* roots are used to treat inflammation and pain [4]. Cannabinoids, abundant in secretory glandular trichomes (GTs) densely inlaid in the female inflorescence of *Cannabis* [5], exhibit medicinal or recreational properties.

Cannabinoids, a group of C21 terpenophenolic compounds, are the most abundant in *Cannabis*. Among them, the well-known psychoactive cannabinoid ∆^9^-tetrahydrocannabinol (Δ^9^-THC) has addictive properties but is efficacious as an analgesic, antiemetic, and antispastic agent. Further, cannabidiol (CBD), a nonpsychoactive cannabinoid, has been clinically validated to treat specific medical conditions, such as epilepsy, glaucoma, and depressive disorder. Moreover, CBD shows potential as an anti-inflammatory treatment for severe acute respiratory syndrome coronavirus 2 (SARS-CoV-2) infection [6]. *Cannabis* has traditionally been classified into two primary types according to the concentration of THC: industrial hemp (THC <0.3%) and marijuana (THC ≥0.3%) [7]. Owing to the high economic value of CBD, an updated classification has emerged for medicinal *Cannabis*, with THC content <0.3% and high CBD levels [8]. However, the legal status and regulations surrounding the use of *Cannabis* and its cannabinoids vary by different countries. Therefore, it is essential to seek guidance from medical professionals and adhere to local laws and regulations when considering the medical applications of *Cannabis*. The global legal *Cannabis* market is projected to reach a value of US$102.2 billion by 2030, driven by the increasing medical and economic demands (https://www.giiresearch.com/publisher/GRVI/). This growth also presents significant challenges for *Cannabis* cultivation and cannabinoid production.

Remarkable advancements in the cultivation of *Cannabis* plants with high-yielding cannabinoids and the reconstruction of cannabinoid production in microorganisms via metabolic engineering have been achieved [9]. The integration of multi-omics methodologies, including genomics, transcriptomics, and metabolomics, has provided comprehensive insights into the genetic composition, gene expression patterns, and regulation of cannabinoid biosynthesis. Further, the relaxation of regulations and the authorization of *Cannabis* research have expanded the opportunities for studying this plant. Here, we summarize *Cannabis* historical usage, cannabinoid biosynthesis, trichome development regulatory network, and cannabinoid metabolic engineering. This review aims to enhance the understanding of *Cannabis* development and utilization, paving the way for further exploration and innovation in *Cannabis* research.

## History and domestication of *cannabis*

### *Cannabis*: ancient significance, enduring influence

*Cannabis* has a long history of human use, with documented evidence from around the world [10, 11] (Fig. S1). The medicinal use of *Cannabis* was first documented in ‘Shen Nong Ben Cao Jing’, which is said to have originated from the emperor Chen Nung (2737 BCE) and was passed down orally until it was assembled (dated no later than 221 BCE). In Egypt, the medicinal use of *Cannabis* was first mentioned in ‘*Papyrus Ramesseum* III’ (1550 BCE), where *Cannabis* was ground and used to treat eye diseases [12]. In India, ‘Soma’ (potentially originating from *Cannabis*) was considered paradisiacal. The description of Soma in *Rig Veda* (1400 BCE), an ancient Indian literature, may be the earliest reference to the psychoactive activity of *Cannabis* [13]. Subsequently, *Cannabis* has been recorded in many important medical texts such as ‘Shi Liao Ben Cao’ and ‘Compendium of Materia Medica’ in China, providing detailed information on the medicinal effects of *Cannabis* inflorescence and seeds. Furthermore, classical medicinal recipes frequently included *Cannabis*, for example, Hua Tuo used *Cannabis* to make ‘Ma Fei San’ for anesthesia in 207 AD, demonstrating the widespread recognition and appreciation of the therapeutic properties of *Cannabis* in the ancient society [14]. A series of recent events on the medicinal properties of *Cannabis* have gradually unraveled its pharmacological mechanism. In 1798, Napoleon brought *Cannabis* back to France from Egypt and investigated it for its pain-relieving and sedative qualities [15]. In the 20th century, CBD and THC were successfully isolated and synthesized in succession [16–18]. Subsequently, the discovery of CB1 and CB2 receptors, the target of cannabinoids in the human body, and endocannabinoid system (ECS) has revealed the relationship between cannabinoids and human health in maintaining homeostasis and influencing various functions such as sleep, appetite, pain perception, inflammation, memory, mood, and reproduction [19, 20].

Historically, *Cannabis* use has encompassed various productive and religious purposes, playing a crucial role in diverse aspects of people’s lives. Originally, *Cannabis* was primarily cultivated to obtain seeds and fiber [21]. Approximately 3000 years ago, ‘The Book of Songs’ provided a detailed record of the entire process involved in hemp clothing production, likely making it the earliest written record. Moreover, the ‘Shuo Wen Jie Zi’ (121 AD) described that disorderly padded hemp fibers were used to keep warm during winter. These ancient written records highlight the significance of hemp as an important textile material at that time. Additionally, increasing archaeological evidence indicated the spiritual use of *Cannabis* smoke before 1260 BCE. It is believed to be the earliest discovery of the narcotic and hallucinogenic effects of *Cannabis* [22]. Cannabinol (CBN), an oxidation product of THC, was detected on the surface of ten wooden fire pots and cobblestones excavated from the Jirzankal cemetery in the Pamir plateau (500 BCE), suggesting the clear evidence of the burning of *Cannabis* [23].

The psychoactive properties of marijuana have led to restrictions on its usage in various countries. In recent times, however, some countries and regions have begun liberalizing the restrictions on *Cannabis* for its economic and pharmacological benefits. California was the first US state to legalize the drug marijuana, ushering in the era of store-bought, commercially made edibles in 1996 [24]. Colorado was the first state to legalize recreational marijuana, imposing a limit of 10 mg THC per serving for edibles in 2014 [25]. In the same year, Uruguay became the first country to legalize *Cannabis*. Subsequently, Thailand was the first Asian country to legalize *Cannabis* in 2022 [26]. The legalization has allowed the development of more diverse properties of *Cannabis* for industrial and medical usage.

### Origin and domestication of *Cannabis*

The origin and domestication of *Cannabis* are debated. Previously, it was widely believed that *Cannabis* originated in Central Asia, supported by the presence of its pollen in India around 32 000 years ago and in Japan around 10 000 BCE [27–29]. Central and Southeast Asia have been proposed as potential regions for its natural origin and primary domestication, playing a significant role in its evolutionary history [30]. However, recent microfossil (fossil pollen) data suggest a center of origin in the northeastern Tibetan Plateau [7]. Further, studies examining the correlation between genetic and geographic distances based on the chloroplast genome propose that *Cannabis* likely originated in low-latitude regions [31]. The complex genetic makeup of *Cannabis*, characterized by high heterozygosity, has posed challenges in studying its domestication history [32]. According to achene fossils found in East Asia and Europe, early humans employed hemp as a fiber plant, whereas the ancient use of the drug marijuana dates back to at least 2700 years ago in Central Asia, as discovered via wooden pots containing THC, which were probably employed for ritualistic and medicinal purposes [23, 33, 34]. However, large-scale whole-genome resequencing studies involving 110 *Cannabis* resources from around the world suggest that *Cannabis* was domesticated in East Asia during the early Neogene [35]. Over millennia, both artificial domestication and wild cultivation led to the development of diverse *Cannabis* species, which are cultivated for their fiber and as drugs (Fig. S1, see online supplementary material).

## Cannabinoids

### Cannabinoids and human health

**Figure 1:**
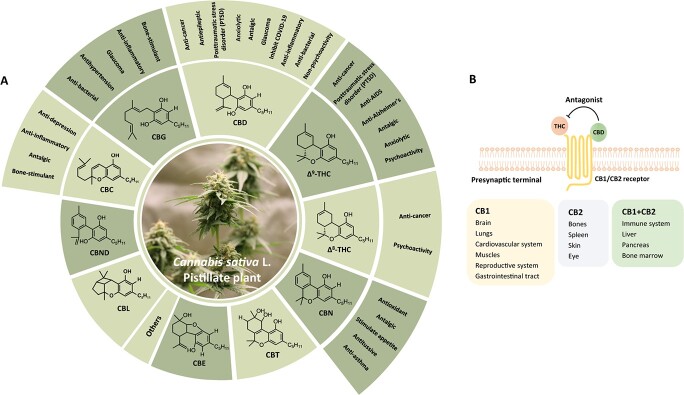
**.** Cannabinoids and human health. **A** Eleven subclasses of cannabinoids in *Cannabis* and their respective pharmacological activities. ∆^9^-THC (∆^9^-tetrahydrocannabinol), ∆^8^-THC (∆^8^-tetrahydrocannabinol), CBG (cannabigerol), CBC (cannabichromene), CBD (cannabidiol), CBND (dehydrocannabidiol), CBE (cannabielsoin), CBL (cannabicyclol), CBN (cannabinol), CBT (dihydroxycannabinol). **B** Cannabinoid receptors CB1/CB2 are distributed in the presynaptic terminal. THC activates the CB1/CB2 cannabinoid receptors, which leads to the release of neurotransmitters, the control of pain perception and memory learning, and the regulation of metabolic and cardiovascular systems. CBD can antagonize THC and reduce its side effects and addiction. Rectangle indicates the distribution of CB1/CB2 cannabinoid receptors in the human body.

*Cannabis* contains numerous natural compounds [36] and over 560 compounds have been identified, such as cannabinoids, phenolics, terpenes, and alkaloids. Cannabinoids form the majority of compounds found in *Cannabis*, with more than 130 cannabinoids having been isolated. They can be classified into eleven subclasses based on their chemical structures, including ∆^9^-THC, ∆^8^-THC, CBG (cannabigerol), CBC (cannabichromene), CBD, CBND (dehydrocannabidiol), CBE (cannabielsoin), CBL (cannabicyclol), CBN, and CBT (dihydroxycannabinol) [37, 38] (Fig. 1A). Cannabinoids exist in two chemical forms: decarboxylated forms and carboxylated forms. In fresh *Cannabis* tissues, carboxylated form is the predominant form. However, through nonenzymatic reactions such as drying, aging, heating, or incineration, *Cannabis* and its extracts undergo decarboxylation of carboxylated forms to form decarboxylated forms [39].

THC and CBD are the most significant compounds in *Cannabis*, and their effects can be perceived as both positive and negative [40]. Clinical pharmacological studies have demonstrated that THC has potent hallucinogenic and addictive properties by activating both CB1 and CB2 receptors, whereas excessive consumption of exogenous cannabinoids can lead to the overstimulation of CB1 and CB2 receptors, causing reduced sensitivity and making it difficult for the body’s natural endocannabinoids to activate them [41, 42]. Thus, some individuals may be more prone to the effects of THC, although it also exhibits antiepileptic, antitumor, and antiemetic effects [43, 44]. In contrast, CBD, a nonpsychoactive component of *Cannabis*, acts as an antagonist to the association between THC and the receptors, effectively mitigating the hallucinogenic effects of THC on the body. CBD can be used in the restoration of the ECS, helping alleviate physical ailments and emotional distress [45, 46] (Fig. 1B). It has verified that CBD possesses diverse pharmacological effects, including antibacterial, anti-inflammatory, anxiolytic, and antiepileptic properties, and it is considered safe for humans [47–49].

Recent studies have revealed promising findings regarding the therapeutic properties of various cannabinoids [6, 50]. For instance, CBC is reported to exert anti-inflammatory, antitumor, antidepressant, and antifungal effects [51–54]. CBG shows potential as an antibiotic and has also been associated with antitumor, antidepressant, analgesic, and glaucoma-alleviating properties [51, 52, 55, 56]. Meanwhile, CBN stimulates appetite and possesses anti-asthma, tranquilizing, and pain-relieving properties [51, 52, 57]. However, some of the lesser-known cannabinoids may be scarce and difficult to extract, resulting in limited research on their medicinal functions and functional activities. Further exploration is needed to fully understand their potential benefits.

### Cannabinoid biosynthesis

**Figure 2:**
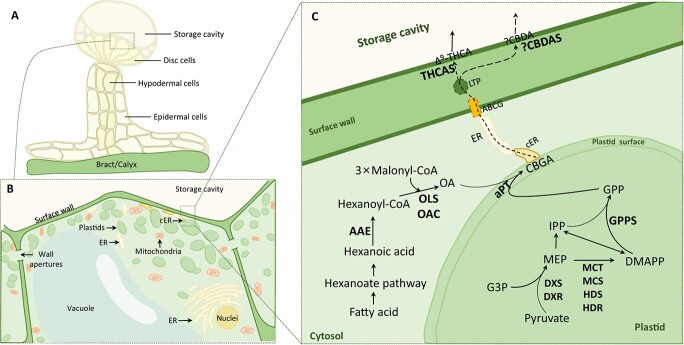
**.** Biosynthesis, trafficking, and secretion of cannabinoids in *Cannabis* glandular trichomes (GTs). **A** Structural diagram of secretory GTs. **B** The distribution of plastids in secretory-stage GT disc cell. Plastids move between cells via apertures in the GT cell wall, aggregating below the surface wall and storage cavity. **C** The biosynthesis and secretion of THCA in GT disc cell. Cytosol-produced olivetolic acid (OA) and plastid-produced geranyl pyrophosphate (GPP) are converted by plastid envelope-localized aromatic prenyltransferase (aPT) into cannabigerolic acid (CBGA). CBGA likely partitions into the plastid membrane bilayer owing to its lipophilicity. Endoplasmic reticulum (ER) membrane contact between the ER and plastids may facilitate lipid metabolite transfer through lipid-binding proteins or transient hemifusions. CBGA may be exported by ATP-binding cassette G-family (ABCG) proteins and transported through the hydrophilic cell wall via lipid transfer proteins (LTPs). Cannabinoid biosynthesis occurs at the disk cell surface wall, where THCA synthase (THCAS) or CBDA synthase (CBDAS) catalyzes CBGA to produce THCA or CBDA, respectively. Cannabinoids form lipophilic metabolite droplets within the apical cell wall, creating storage cavity space. Abbreviations: DMAPP, dimethylallyl pyrophosphate; DXR, deoxyxylulose phosphate reductoisomerase; DXS, deoxyxylulose phosphate synthase; G3P, glyceraldehyde 3-phosphate; GPPS, geranylpyrophosphate synthase; HDR, hydroxymethylbutenyl diphosphate reductase; HDS, hydroxymethylbutenyl diphosphate synthase; IPP, isopentenyl diphosphate; MCS, methylerythritol cyclodiphosphate synthase; MCT, methylerythritol phosphate cytidylyltransferase; MEP, methylerythritol phosphate.

The secretory GTs are known as the ‘plant chemical factory’, secreting and storing multiple secondary metabolites (Fig. 2A). Cannabinoids are synthesized and accumulated in the *Cannabis* secretory GTs, and the content of cannabinoid is related to the secretion, type, and density of GTs [5]. Until now, the skeleton biosynthesis of cannabinoids has been elucidated, including two main biosynthetic pathways (Fig. 2B and C). The first pathway is polyketide synthesis occurring within the cytosol. It involves the conversion of hexanoic acid to thiolate hexanoyl coenzyme A by acyl-activating enzyme (AAE) [58]. Olivetol synthase (OLS) then catalyzes hexanoyl coenzyme A condensation with malonyl coenzyme A, leading to the formation of an intermediate tetraketide-CoA [59]. This intermediate is further cyclized by olivetolic acid cyclase (OAC) to produce olivetolic acid (OA), which serves as the polyketide nucleation component required for cannabinoid synthesis [60, 61]. The second pathway is the methylerythritol 4-phosphate (MEP) pathway, which generates isopentenyl diphosphate and dimethyl allyl diphosphate. These two compounds combine to form geranyl pyrophosphate (GPP), an isoprenoid compound that provides the monoterpene fraction needed for cannabinoid biosynthesis [40]. The GPP biosynthesis occurs in the plastid matrix, where it can freely move within the hydrophobic membrane [62]. Furthermore, on the plasma membrane, GPP and OA are catalyzed into cannabigerolic acid (CBGA) via the plastid envelope-localized aromatic prenyltransferase (aPT), known as CBGAS [63]. CBGA serves as a common precursor of partial oxidative cyclization to tetrahydrocannabinolic acid (THCA), cannabichromenic acid (CBCA), and cannabidiolic acid (CBDA), which is catalyzed by THCA synthase (THCAS), CBCA synthase, and CBDA synthase (CBDAS) [64–66]. The final step in cannabinoid biosynthesis occurs on the cell wall surface in the extracellular storage lumen [67]. The acidic forms of Δ^9^-THCA, CBCA, and CBDA are then converted to THC, CBC, and CBD, respectively, through a nonenzymatic decarboxylation process. Other cannabinoids can be synthesized through isomerization from THC, CBD, and CBC, and conversions between cannabinoids can also occur under specific conditions [34].

With advancements in sequencing technology, multiple versions of the *Cannabis* genome have been published, providing a deeper understanding of the synthesis mechanism of THC and CBD from CBGA at the genomic level. Grassa *et al.* sequenced the *Cannabis* genome and identified the specific location of THCAS and CBDAS on chromosome 9 [68]. Subsequent studies using updated genome versions further examined THCAS and CBDAS in two *Cannabis* strains (PK and FN) and revealed significant rearrangements of motifs related to THC/CBD synthesis [32, 69]. Further, CBDAS has a higher affinity for CBGA than that for THCAS. Consequently, when both enzymes are present, the production of CBDA is favored, suggesting why *Cannabis* plants typically contain higher CBD levels than THC levels [70]. Initially, THCAS and CBDAS were considered as an allele pair [71]. However, de Meijer *et al.* found that the THCA/CBDA ratio in medical marijuana F1 plants followed a Mendelian expectation of 1:2:1 [72]. This observation, along with the identification of major quantitative trait loci, suggested the presence of a single locus that exhibits codominant Mendelian inheritance patterns [73]. However, Kojoma *et al.* evidenced the presence of THCAS and CBDAS genes at two distinct loci on the genome that are linked together [74]. These findings have complicated our understanding of the genetic basis of THC and CBD synthesis in *Cannabis*.

Whole-genome resequencing of *Cannabis* revealed that nearly all drug *Cannabis* samples contained the complete coding sequence of THCAS and two CBDAS pseudogenes, while the majority of fiber *Cannabis* samples only had the full CBDAS-encoding gene [35], suggesting that both genes were initially present and functional in the ancestral state. Early domestication involved polymorphism and random loss of function in one of the genes, with THCAS and CBDAS losing their functions in hemp and in drug *Cannabis*, respectively, after strong artificial selection. The competitive relationship between these two genes in cannabinoid synthesis likely contributed to their loss of function, which was associated with the increase and decrease in THC content in drug *Cannabis* and in industrial hemp, respectively. Although the *de novo* sequencing and resequencing of the *Cannabis* genomes provided insights into the genetic basis and localization of genes involved in cannabinoid biosynthesis, further research is needed to explore the genomic evolution, biosynthesis, and regulation of target compounds. A deeper understanding at the chromosomal level as well as genome-wide association studies (GWAS) will contribute to a more comprehensive understanding of these aspects.

### Regulatory network of cannabinoid biosynthesis

Manipulation of external environmental factors is a significant area for increasing the cannabinoid content like CBD and decreasing the content of THC. Exogenous hormone treatments, including gibberellic acid, methyl jasmonate, and salicylic acid, influence cannabinoid synthesis in *Cannabis* [75–79]. Additionally, the use of mevinolin can effectively reduce THC content by inhibiting the MEP and mevalonate pathways [75]. Polyploidization treatments have increased CBD content without altering THC accumulation [80, 81]. Furthermore, light conditions differently affect the synthesis of different cannabinoids [79, 82, 83]. These findings highlight the potential of manipulating external factors to modulate cannabinoid composition in *Cannabis*. Additionally, transcription factors (TFs) have been identified as key regulators that activate or inhibit the biosynthesis of plant natural products, effectively enhancing the synthesis of desired secondary metabolites. Numerous reports have highlighted the significance of TFs in regulating secondary metabolite synthesis [84–87]. To date, many TF gene families, such as WRKY, HD-ZIP, MYB, and bHLH, have been identified as being related to cannabinoid biosynthetic regulation based on genome-wide strategies in *Cannabis* [88–91]. However, the lack of a robust genetic transformation system hinders comprehensive studies on the mechanisms regulating cannabinoid biosynthesis.

Plant genetic transformation allows plants to acquire new traits, contributing significantly to variety improvement and gene function elucidation. However, for *Cannabis* as a regenerative and genetically recalcitrant plant, it is difficult to develop an efficient genetic transformation system, with low regeneration efficiency influenced by factors such as variety, tissue type, plant age, and growth regulator combination [92, 93]. Previous studies employed different methods, such as *Agrobacterium*-mediated transformation, virus-induced gene silencing, and nanomaterial translocation, to achieve transient expression in *Cannabis* leaves, albeit with limited efficiency [94–99]. Recently, efforts have been made to develop stable genetic transformation systems for *Cannabis*. Wahby *et al.* successfully induced transgenic hairy roots; however, cannabinoids were not detected, possibly owing to their accumulation primarily in GTs rather than that in roots [100]. Therefore, it is not feasible to study the cannabinoid biosynthesis regulation through transgenic hairy roots. Zhang *et al.* developed transgenic plants through *Agrobacterium*-mediated infection but with low redifferentiation efficiency (≤7.09%) and a high proportion of chimeras [101]. Further, Galán-Ávila *et al.* optimized the transgenic system and successfully induced regenerated seedlings directly from hypocotyls or cotyledons [102]. Nevertheless, the establishment of an efficient transgenic system, for both transient and stable transformation, remains a significant challenge in *Cannabis* research, particularly for investigating cannabinoid synthesis regulation and other important traits.

## Glandular trichomes in *Cannabis* and their potential transcriptional regulatory network

GTs arise from the differentiation of epidermal cells and are prominent on the surface of numerous plant species [103]. GTs are important defense organs against environmental stress, while also offering significant economic and practical value through their specialized metabolites [104–106]. Secretory GTs are specifically involved in the synthesis, accumulation, and release of a wide range of metabolites, including organic acids, polysaccharides, polyphenols, flavonoids, alkaloids, and terpenoids [107–109]. In contrast, nonsecretory GTs primarily function as protective structures without chemical compound secretion [110].

**Figure 3:**
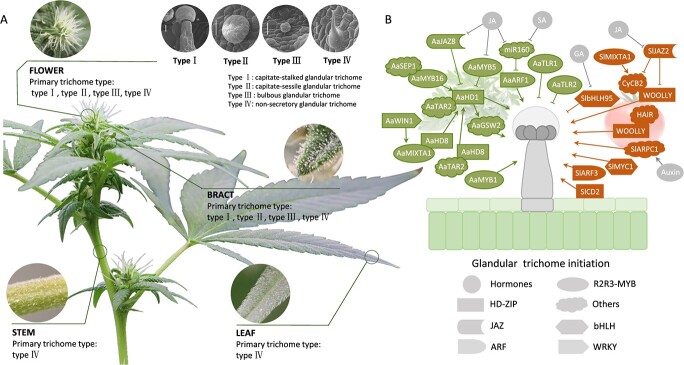
**.** GTs in *Cannabis* and their transcriptional regulatory network in *Solanum lycopersicum* and *Artemisia annua*. **A** Scanning electron microscopy analysis illustrating the different GT types on the surface of various tissues in *Cannabis sativa*. Type I, capitate-stalked GT; Type II, capitate-sessile GT; Type III, bulbous GT; Type IV, nonsecretory GT. Bars = 25 μm. **B** Summarized transcriptional regulatory network of GT initiation in *S. lycopersicum* and *A. annua*. Different shapes represent distinct regulatory factors. Arrow-headed lines indicate upregulation, whereas blunted lines indicate downregulation or inhibition.

Secretory GTs are abundantly present on the tissue surface of multiple plants such as Solanaceae, Labiatae and Asteraceae. Given their similar structure, the secretory GTs may have similar developmental events in different plants species [111]. *Arabidopsis thaliana* only possesses nonsecretory GTs [112, 113]. Consequently, although more comprehensive studies on the morphological aspects, density, and developmental processes of GTs have been conducted on *Arabidopsis*, the mechanisms underlying the development and specialized metabolite biosynthesis in secretory GTs may not be fully elucidated by using the model plant *A. thaliana*. On the contrary, the recently extensive studies in *Artemisia annua* and *Solanum lycopersicum* exemplify the transcription factors regulating secretory GTs development. Therefore, we summarized a transcriptional regulatory network of GTs development in these both species in the later part of this review to provide a reference for the regulation of GTs in *Cannabis* (Fig. 3).

### Characteristics of glandular trichomes in *Cannabis*

*Cannabis* GTs are distinctive secretory structures that are crucial in the production and reservation of cannabinoids, primarily found on the bracts and flowers of female *Cannabis* plants (Fig. 2A) [114]. In *Cannabis*, the density of GTs per unit area is generally higher in female plants than that in male ones [115]. Female plants exhibit a greater abundance of GTs on bracts, flowers, and other plant parts (Fig. 3A). GTs in *Cannabis* can be classified into two main types: secretory GTs and nonsecretory GTs [116]. Secretory GTs are further categorized into stalked GTs, sessile GTs, and bulbous GTs [5]. Stalked GTs consist of a stalked base with 12–16 secretory disc cells; sessile GTs possess a short-stalked base with eight secretory disc cells; and bulbous GTs are the smallest in size and consist of a short-stalk base with a bulbous head. Additionally, *Cannabis* plants possess two types of nonsecretory GTs, which primarily serve as a protective barrier in the plant’s epidermis, shielding it from external damage [116, 117]. The type and density of secretory GTs exhibit variations during development. In the early developmental stages in bract and flower, all GT types are present, whereas in the later stages stalked GTs are predominant, indicating that sessile GTs may represent an early stage in stalked GT development [5].

The synthesis and storage of cannabinoids primarily occur in secretory GTs [118]. Through the isolation and analysis of *Cannabis* GTs, Happyana *et al.* confirmed that both bulbous GTs and stalked GTs can produce CBGA and THCA. Additionally, CBC and CBN were specifically detected in bulbous stalked GTs [119]. Autofluorescence studies further supported these findings and revealed the higher accumulation of monoterpenes in the stalked GTs [5]. The analysis of *Cannabis* GT wall components revealed the presence of loosely bound xyloglucan and pectin polysaccharides. Within the cell wall, the interaction between polysaccharide emulsions and metabolite droplets creates a crucial microenvironment where the boundary between metabolites and polysaccharides acts as a hydrophobic–hydrophilic interface [120]. Cannabinoid synthases have greater metabolic efficiency in hydrophobic environments than that in aqueous ones [118]. This metabolite–polysaccharide wall boundary may be a favorable microenvironment that is well suited for THCA biosynthesis, which could explain the abundant synthesis and secretion of cannabinoids in GTs [120]. Despite their small size, the ability of these GTs to produce and secrete significant quantities of secondary metabolites remains a mystery. Unraveling the mechanisms behind their metabolite synthesis should provide valuable insights for improving the production capacity of natural products in microorganisms and plant chassis.

Furthermore, ultra-rapid cryofixation, quantitative electron microscopy, and immuno-gold labeling of enzymes involved in the cannabinoid pathway revealed that metabolically active cells in *Cannabis* GTs form a specialized ‘supercell’ [67, 121]. These supercells exhibit extensive cytoplasmic bridges across their cell walls and a polarized distribution of organelles adjacent to the apical surface, which is responsible for metabolite secretion. These supercells are organized as polarized cell syncytia, representing a synchronized fusion of multiple cells with no discernible cytoplasmic connection to the underlying tissue. This polarized cell syncytium configuration presents a potential mechanism for preventing the backflow of metabolites from the trichomes by physically isolating the syncytium from the surrounding tissue.

### Transcriptional regulation in the development of secretory GTs

The regulatory mechanisms governing the GT development in *Cannabis* are not yet fully understood. However, the *MIXTA* gene, an MYB family transcription factor, has been identified as a regulator of multicellular epidermal hairs and conical cells in the leaves of *Antirrhinum majus* [122, 123]. Attempts to overexpress the *MIXTA1* homolog from *Cannabis* in tobacco led to an increase in GTs [124]. To date, the regulatory mechanisms underlying the development of secretory GTs in *S. lycopersicum* and *A. annua* have been extensively studied. Herein, we summarize the progress in research on TFs and noncoding RNAs that regulate the development of secretory GTs.

### Molecular mechanism of secretory GT development in *S. lycopersicum*

Several TFs involved in the development and morphology of GTs in tomatoes have been identified. Chang *et al.* discovered a C2H2 zinc finger protein gene, *Hair*, associated with the absence of GTs using GWAS analysis [125]. Transgenic experiments showed that the hair-absent phenotype was caused by deletion of the entire coding region of *Hair*. Additionally, *Hair* was found to interact with WOOLLY to promote GT development [126]. Further, Chun *et al.* explored the transcriptome differences between *hairless* mutants and the wild type and discovered that downregulation of the actin-related protein component 1 (SlARPC1) led to the severe curvature of GTs in *hairless* mutants [127]. *SlCycB2*, encoding a B-type cell cycle protein, reduces the secretory GT density. Further study revealed that *SlCycB2* expression was increased in plants overexpressing *WOOLLY* and *SlMIXTA1*, whereas it was reduced in *woolly* mutant plants [128]. Yu *et al.* discovered that *SlJAZ2* inhibited GT development by suppressing *WOOLLY* and *SlCycB2* activity [129]. An ARF family transcription factor, *SlARF3*, highly expressed in GTs, positively regulates GT initiation [130]. *SlMYC1* is also involved in the initiation and morphogenesis of secretory GTs. Knockdown of *SlMYC1* led to smaller and less dense trichomes, whereas its knockout resulted in the disappearance of secretory GTs. Furthermore, Chen *et al.* discovered that *SlbHLH95* suppressed GT development by repressing the expression of key genes (*SlGA20ox2* and *SlKS5*) involved in endogenous gibberellic acid synthesis [131]. Overall, these tomato TFs related to GT development have provided valuable insights into the genetic mechanisms underlying the development and regulation of GTs in *Cannabis* (Fig. 3B).

### Molecular mechanism of GT development in *A. annua*

The World Health Organization recommends artemisinin as an essential component of standard antimalarial therapy, with *A. annua* being its unique natural plant source. Artemisinin is specifically distributed in the secretory GTs in *A. annua*, and the developmental transcriptional regulatory networks of these trichomes have been extensively investigated. *AaHD1*, an HD-ZIP family transcription factor, as a key regulator of GT initiation in *A. annua*, has functions in increasing trichome density and artemisinin production [132]. *AaJAZ8* antagonizes *AaHD1* expression and suppress GT development. Another HD-ZIP family TF, *AaHD8*, promotes *AaHD1* expression and trichome initiation. The overexpression of *AaGSW2*, a gene specifically expressed in GTs and coexpressed with *AaHD1*, increased trichome density [132]. *AaMIXTA1*, a homologous gene of the positive regulator MIXTA, is also involved in regulating GT development by binding to the *AaHD8* promoter [133]. The AP2/ERF family TF AaWIN1 interacts with AaMIXTA1 to induce *AaGSW2* and promote trichome development [134]. *AaMYB16*, another homolog of *AaMIXTA-like2*, is bound to the L1-box on the *AaHD1* promoter to enhance *AaHD1* expression, whereas AaMYB5 is competitively bound to the *AaHD1* promoter by binding to AaJAZ8, exerting a repressive effect [135]. The MADS-box family TF AaSEPALLATA1 (AaSEP1) enhances the regulatory effect of AaHD1 on AaGSW2 by binding to AaMYB16, thereby regulating trichome development [136]. These identified TFs indirectly or directly regulate *AaHD1* expression, forming an AaHD1-centered regulatory network that serves as a reference for studies on GT initiation. Other MYB family TFs like MYB1, TrichomeLess Regulator 1 (TLR1), and TLR2 also regulate GT initiation [137, 138].

MicroRNAs (miRNAs) are crucial in regulating terpenoid biosynthesis by targeting key enzymes and TFs involved in secondary metabolite synthesis. Overexpression of miR160 leads to significant inhibition of GT initiation and artemisinin biosynthesis [139]. Conversely, inhibition of miR160 expression had the opposite effect, indicating that miR160 negatively regulates the GT initiation and artemisinin biosynthesis. This regulatory mechanism is mediated by miR160’s cleavage of AaARF1, providing valuable insights into the transcriptional regulatory network governing GT synthesis in *A. annua* (Fig. 3B).

Based on the progress in research on the TF-mediated regulation of secretory GTs in tomato and *A. annua*, the homologous genes in *Cannabis* and/or the trichome-specific TFs could be identified as a potential transcriptional regulatory network associated with GT development in *Cannabis*. Furthermore, the regulatory mechanisms of candidate TFs related to cannabinoid biosynthesis and secretory GT initiation need to be further elucidated by *in vivo* and *in vitro* biochemical strategies. Furthermore, single-cell transcriptome sequencing technology could be used to identify genes specifically expressed in *Cannabis* secretory GTs, providing an important reference for precise mining of regulatory genes related to *Cannabis* secretory GT development and cannabinoid biosynthesis [140, 141].

## Advances in metabolic engineering of cannabinoids

At present, Δ^9^-THC and CBD, the primary compounds of interest, are extracted directly from *Cannabis*. However, the study and medicinal use of cannabinoids have faced challenges owing to legal restrictions on *Cannabis* and the low natural abundance of most cannabinoids. In addition, the complex structures of cannabinoids hinder their large-scale chemical synthesis. Green bioproduction of cannabinoids based on the theory of synthetic biology could efficiently overcome these limitations by providing a comprehensive understanding of the cannabinoid synthesis pathway, synthesizing and assembling the essential components in microbial cells, and optimizing the fermentation production of target cannabinoids.

### OA production

CBGA, formed through the condensation of OA and GPP, is the primary precursor molecule in cannabinoid biosynthesis. Therefore, achieving high levels of OA production is crucial for effective metabolic engineering of cannabinoids.

*Escherichia coli* is widely recognized as an ideal host for microbial production owing to its straightforward genetic background and rapid reproduction [142]. Tan *et al.* successfully achieved OA synthesis in *E. coli* by coexpressing *OLS* and *OAC* with the required modules of a *β*-oxidation reversal for hexanoyl-CoA generation. Furthermore, the integration of supplementary enzymes to enhance the production of hexanoyl-CoA and malonyl-CoA, along with the evaluation of varying fermentation conditions, enabled the synthesis of 80 mg/L OA, marking a significant milestone as the first reported production of OA in *E. coli* [143].

By introducing a group of fungal tandem polyketide synthases in the model fungus *Aspergillus globulus*, OA was produced at high yields of up to 80 mg/L. This finding presents a novel and alternative method for the microbial production of cannabinoid precursors, bypassing the need for the OLS and OAC enzymes derived from *Cannabis* [144].

*Yarrowia lipolytica*, a safe and lipid-rich yeast, has been successfully engineered to produce various valuable natural products [145, 146]. Introduction of LvaE from *Pseudomonas* sp*.*, which encodes a short-chain acyl-CoA synthetase, enables efficient conversion of hexanoic acid to hexanoyl-CoA in *Y. lipolytica* via coexpressing acetyl-CoA carboxylase, the pyruvate dehydrogenase bypass, the NADPH-generating malic enzyme, as well as activation of the peroxisomal *β*-oxidation pathway and ATP export pathway. These modifications effectively redirect the carbon flux toward OA production. The optimized protocols led to a remarkable 83-fold increase in OA production, with a titer of 9.18 mg/L, providing an excellent microbial chassis for high-throughput production of cannabinoids [147].

In contrast to fungi and plants, amoebae represent a unique group of eukaryotes that express terpene synthases, indicating the presence of an active GPP biosynthesis pathway [148]. Reimer *et al.* utilized the amoeba *Dictyostelium discoideum* as a host organism to successfully produce phlorocaprophenone, methyl-olivetol, resveratrol, and OA by sequentially expressing native cannabinoid polyketide synthase. To facilitate OA synthesis, an amoeba/plant inter-kingdom hybrid enzyme was further engineered to produce OA from primary metabolites by only two enzymes, thus providing a shortcut to the synthetic cannabinoid pathway using the *D. discoideum* chassis [149]. Subsequently, the optimization of reaction conditions in *D. discoideum* led to OA production with productivity of 0.04 μg/L/h and a concentration of 4.8 μg/L by scaling up the bioreactor to an industrial scale of 300 L [150].

### *De novo* cannabinoid synthesis

*De novo* and high-yield production of cannabinoids in microorganisms or heterogenous plants have been gaining attention for its global demand. Luo *et al.* first achieved *de novo* bioproduction of cannabinoids in yeast via the introduction of OA biosynthetic modules, optimization of the mevalonate pathway, and the identification of novel prenyltransferase and different cannabinoid synthases [63]. The exogenous hexanoyl-CoA enzymes including *RebktB*, *CnpaaH1*, *Cacrt*, and *Tdter* [151], combined with *Cannabis* CsTKS and CsOAC, improved the yield of OA to 1.6 mg/L. Further, site-direction mutations of ERG20^F96W/N127W^ for increasing GPP accumulation, and the identification and introduction of novel *Cannabis* CsaPT4 in yeast led to the successful synthesis of 7.2 mg/L and 1.4 mg/L CBGA by adding galactose and 1 mM hexanoic acid and by adding galactose, respectively. Furthermore, the replacement of N-terminal secretory signal peptides of THCAS and CBDAS with a vacuolar localization tag led to yields of THCA and CBDA of 2.3 mg/L and 4.2 μg/L, respectively. Finally, by increasing the copy numbers of the key enzymes *CsTKS*, *CsOAC*, *CsaPT4*, and *THCAS*, the THCA yield reached 8 mg/L. Although this yield is only at the milligram level, it demonstrates the potential of *Saccharomyces cerevisiae* as a microbial chassis for cannabinoid synthesis. Qiu *et al.* further *de novo*-synthesized CBD in *S. cerevisiae* by integrating the CBD biosynthetic gene modules. In addition, the transporter protein BPT1 could effectively transfer CBGA from cytoplasm to vacuole, and the combination of BPT1 in engineered yeast improved the CBD content to 6.92 mg/L [152].

**Table 1:** Biosynthesis of OA and cannabinoids using metabolic engineering

**Organism**	**Methods**	**Products**	**Yield**	**Reference**
*Yarrowia lipolytica*	Used a short-chain acyl-CoA synthetase co-expressed with the acetyl-CoA carboxylase	Olivetolic acid (OA)	9.18 mg/L	Ma *et al.* (2022) [147]
*Escherichia coli*	Co-expressed *olivetol synthase (OLS)* and *olivetolic acid cyclase (OAC)*, with the module required for *β*-oxidation to reverse hexanoyl-CoA, hexanoyl-CoA and malonyl-CoA	OA	80 mg/L	Tan *et al.* (2018) [143]
*Aspergillus globulus*	Discovered a set of fungal tandem polyketide synthases without relying on the *OLS* and *OAC* found in *Cannabis*	OA	80 mg/L	Okorafor *et al.* (2021) [144]
*Dictyostelium discoideum*	Sequentially expressed native cannabinoid polyketide synthase and introduced an amoeba/plant inter-kingdom hybrid enzyme	OA	4.5 μg/L	Reimer *et al.* (2022) [149]
*Pichia pastoris*	Introduced *tetrahydrocannabinolic acid synthase (THCAS)* in *Pichia pastoris*	Tetrahydrocannabinolic acid (THCA)		Zirpel *et al.* (2015) [157]
*K. phaffii*	Used *NphB* replace the function of *THCAS*,	THCA	82 ± 4.6 pmol/L	Zirpel *et al.* (2017) [153]
*Saccharomyces cerevisiae*	Introduced and modified more than 15 genes from different species into yeast	THCA, Tetrahydrocannabivarinic acid (THCVA)	8 mg/L, 4.8 mg/L	Luo *et al.* (2019) [63]
*S. cerevisiae*	Integrated the CBD biosynthetic gene modules and the transporter protein BPT1	Cannabidiol (CBD)	6.92 mg/L	Qiu *et al.* (2022) [152]
*Nicotiana benthamiana*	Introduced acyl-activating enzyme and OLS	Cannabinoid derivatives		Reddy *et al.* (2022) [156]
*N. benthamiana*	Transient Expression of *CsaPT4*, *SrUGT71E1* and *OsUGT5*	Cannabigerolic acid (CBGA), olivetolic acid glucoside and cannabigerolic acid glucoside		Gulck *et al.* (2020) [155]
	Obtained more active enzyme OAC^F24I^ and TKS^L190G^	different acyl groups on C-3 of OA		Lee *et al.* (2022) [158]
	Obtained more active enzyme NphB^V49W/Y288P^	CBGA		Lim *et al.* (2022) [154]
Cell-free	Constructed a 23-enzyme reaction system	CBGA	1.25 g/L	Valliere *et al.* (2019) [159]
Cell-free	Constructed a 12-enzyme reaction system	CBGA	570 ± 60 mg/L	Valliere *et al.* (2020) [160]

NphB, a prenyltransferase from *Streptomyces* sp., has been ever served as an enzyme to catalyze the production of CBGA [153], and the nonspecific regioselectivity of NphB greatly limits cannabinoid synthesis. Therefore, Lim *et al.* modified and optimized the NphB^V49W/Y288P^ variants, leading to a 13.6-fold improvement in CBGA yield [154]. The cannabinoid synthesis titer in yeast was mainly restricted by the enzymatic activities related to the OA pathway; there is thus a need for further exploitation of the modification and optimization of these enzymes.

*Nicotiana benthamiana* is a potential plant chassis to efficiently synthesize cannabinoids. Thies *et al.* introduced 12 aPTs into *N. benthamiana* and found that CsaPT4 can convert GPP and OA into GBGA. However, OA and its glycosylation products were detected owing to the presence of endogenous glycosyltransferase in engineered *N. benthamiana* [155]. Reddy *et al.* also engineered *N. benthamiana* to synthesize cannabinoid precursors via the stable expression of *AAE* and *OLS*, and transient expression of *OAC* genes. Owing to the endogenous modifications of cannabinoid intermediates in tobacco, the chassis may be further directly engineered, especially the gene silencing or knockdown of modified enzymes, to make it applicable for cannabinoid bioproduction [156].

### Cell-free system for cannabinoid engineering

Microbial production offers an alternative to natural extraction for prenylated cannabinoids but has challenges such as carbon flux diversion, product toxicity, and GPP essentiality, which can hinder large-scale production. Valliere *et al.* developed a cell-free platform to enhance the prenylation of natural products, with a focus on cannabinoid production [159]. Through the introduction of the pyruvate dehydrogenase bypass and optimization of cofactors, enzymes, and environmental factors, the cell-free system achieved CBGA titers of 132 mg/L. Further advancements were made by redesigning the NphB prenyltransferase, resulting in a CBGA titer of 600 mg/L. To enhance enzyme stability, a flow system for CBGA capture was implemented, leading to a titer of 1.25 g/L, representing a 140-fold improvement. The cell-free system was further optimized and designed, optimizing the number of enzymes to only 12 enzymes to produce CBGA, and using the relatively inexpensive substrate acetyl-CoA. The final optimized system could produce 570 ± 60 mg/L CBGA, which was lower than that of the previous system, but the cost was substantially reduced; with the subsequent optimization, the yield could be further increased [160]. These solutions were rapidly achieved through iterative design-build-test cycles, surpassing previously reported results using living cells. However, the large-scale construction of highly complex systems involving numerous enzymes, cofactors, and metabolites using a cell-free system remains a challenge.

The utilization of metabolic engineering to produce cannabinoids offers unparalleled advantages compared with both plant extracts and chemical synthesis. Although significant efforts have been made to produce cannabinoids using various chassis cells such as *E. coli*, yeast, and tobacco, the resulting yields remain considerably low and fall short of the requirements for industrial production (Table 1). Considerable efforts are still required to enhance synthetic efficiency, including the improvement of metabolic flux into the cannabinoid biosynthesis in the chassis, exploration and modification of efficient synthetic enzymes, and optimization of fermentation processes. With the global changes in the legalization of *Cannabis*, further expansion in the market for green cannabinoid bioproduction is expected.

**Figure 4:**
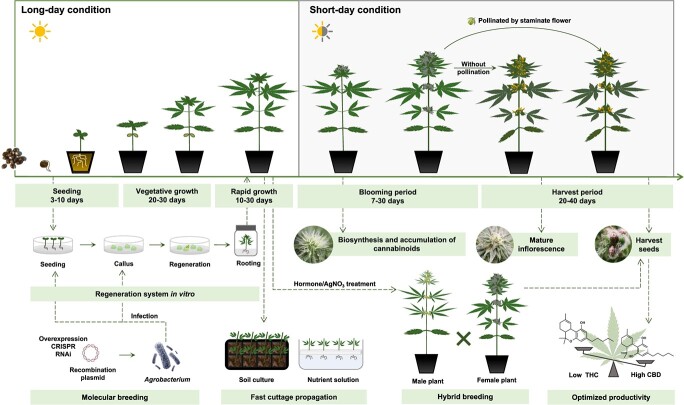
*Cannabis* growth and experimental timelines. *Cannabis* exhibits a relatively short growth cycle, lasting 2–5 months. The seedling stage takes approximately 3–10 days after germination. Subsequently, it enters the vegetative growth stage characterized by slow growth and nutrient absorption focused on root development, lasting for approximately 20–30 days. The subsequently rapid growth of the aerial part is maintained for 10–30 days. Preflowering shoots as cuttings are suitable for asexual propagation. Callus induction and genetic transformation can be performed by taking explants from the hypocotyledonary axis, cotyledon, and tender leaf. The flowering initiation in *Cannabis* can be induced by subjecting the plants to short-day conditions. After approximately a week, flowers begin to appear at the top of the branches. The flowering phase typically lasts for a month. When treated with hormones or AgNO_3_, female plants can be transformed into male plants. The harvesting of inflorescences for cannabinoid extraction can be performed once the pistils have withered. If female flowers are pollinated during the flowering phase, seeds can be collected after approximately a month. New varieties with high CBD yield can be obtained through hybridization breeding or molecular breeding techniques.

## Conclusion and future perspectives

*Cannabis*, an economically important crop, is restricted or banned for cultivation in many countries owing to it containing the psychoactive compound THC. However, the significant medicinal value of various cannabinoids, such as CBD, found in *Cannabis* has led to increased demand for cannabinoids, accelerating the legalization of industrial *Cannabis* cultivation globally. Nevertheless, the lack of comprehensive research on *Cannabis* GT development and cannabinoid biosynthesis regulation poses major challenges for high CBD and low THC *Cannabis* varieties breeding and efficient green bioproduction of cannabinoids. To address these issues and provide crucial support for the globalization of the *Cannabis* industry, there is an urgent need for innovative interdisciplinary and technological research on *Cannabis*. Recently, metabolic engineering of cannabinoids and the cultivation of new medicinal *Cannabis* varieties with high CBD and low THC content have gained importance.

### Understanding the regulatory mechanism of cannabinoid biosynthesis and secretory GTs

TFs, noncoding RNAs, and epigenetic modifications have been identified as key players in plant development and responses to abiotic/biotic stresses, and the biosynthesis of valuable natural products [85, 161]. Cannabinoid biosynthetic genes have been fully elucidated, and they present trichome-specific accumulation. However, the mechanisms regulating cannabinoid biosynthesis and secretory GTs remain largely unclear. Although the regulation of secretory GT development in tomato and *A. annua* could provide homologous evidence on the *Cannabis* trichome initiation, further exploration of *Cannabis*-specific cannabinoid biosynthesis and trichome development are necessary. With the advancements in sequencing technologies, multi-omics data for *Cannabis*, including the T2T genome, 3D genome architecture and epigenomes for flower or trichomes, single-cell transcriptomes, and transcriptomes and metabolomes of different tissues, will provide important genetic resources for the selection of candidate TFs and noncoding RNAs related to cannabinoid biosynthesis regulation and secretory GT initiation [162, 163].

### Multi-omics-guided breeding and cultivation of novel *Cannabis* varieties

*Cannabis* varieties cultured mainly by conventional breeding such as hybridization and systematic selection, and some varieties with high CBD content have been cultured by hybridization, such as ACDC, Harle-Tsu, and Corazón (https://www.leafly.com/news/strains-products/high-cbd-marijuana-strains-according-tolab-data). However, conventional breeding often involves lengthy selection cycles and is less efficient [71]. In contrast, molecular breeding offers a more efficient and precise approach, thereby accelerating the breeding process [164]. Herbgenomics lays the foundation for molecular genetics of medicinal plants. The integration of multi-omics technology has provided additional genetic information for molecular breeding in *Cannabis*. Combining multiple published versions of the *Cannabis* genomes with multi-omics approaches with the aid of artificial intelligence-based data analysis tools has significantly improved the efficiency with which the genetic and molecular mechanisms regulating cannabinoid biosynthesis can be uncovered. Particularly, the relationship between the expression of key enzymes involved in cannabinoid biosynthesis and the THC:CBD ratio and cannabinoid content can be better understood at the genetic and molecular levels. Other breeding strategies such as GWAS use natural populations to identify molecular markers associated with important traits in *Cannabis* [165]. Moreover, GWAS enables the discovery of molecular markers and facilitates the identification of loci and candidate genes related to crucial agronomic traits such as CBD and THC biosynthesis, sexual development, and cellulose quality [166, 167]. Constructing a genetic linkage map of *Cannabis* using molecular marker technology, leveraging the genetic information from diverse genetic resources, and developing molecular markers for desirable traits will further advance the process of *Cannabis* breeding [168, 169].

### Efficient green bioproduction of cannabinoids

Although significant progress has been made in the *de novo* synthesis of cannabinoids in yeast, achieving high yields of cannabinoids is still a major challenge. The rate-limiting steps and metabolic bottlenecks in cannabinoid biosynthetic pathways within chassis cells need to be further addressed and optimized to improve the overall efficiency and yield of cannabinoid production. Site-directed mutations of crucial rate-limiting enzymes in cannabinoid biosynthesis could efficiently improve the yields in chassis cells. Cannabinoid accumulation via parallel evolution has been discovered in *Helichrysum umbraculigerum*, providing a set of alternative enzymes for the synthetic biology of cannabinoids [170, 171]. Further studies can explore the construction of fusion proteins from different plant sources to enhance catalytic efficiency. In addition to microbial and plant chassis, OA synthesis has also been reported in *D. discoideum*. Amoebas offer a wider and larger gene repertoire for polyketide and terpenoid biosynthesis, presenting a new approach for the synthetic chassis of cannabinoids [172].

### Robust model research system for medicinal plants

Since the late Northern Wei Dynasty, detailed techniques for hemp cultivation have been recorded in ‘Qi Min Yao Shu’. In recent times, research on efficient *Cannabis* cultivation has gained significant attention owing to the substantial market demand. Dioecious *Cannabis* offers several advantages as a robust model research system for understanding the development of secretory GTs. *Cannabis* has a relatively short growth cycle of approximately three months, and short-day conditions can induce *Cannabis* flowering initiation. The generation time can be shortened within nine weeks by controlling light condition [173]. Here, we construct a schematic to describe the *Cannabis* growth cycle and corresponding biotechnologically experimental timeline (Fig. 4). Briefly, multiple methods developed and exploited for the genetic transformation of *Cannabis* generally occur at or before seedlings. Fast cutting propagation usually takes place in the rapid growth stage before inflorescence [174]. To avoid genetic heterozygosity introduced by hybrid breeding, as *Cannabis* is dioecious, female plants can be transformed to produce male flowers by treating with hormones or AgNO_3_ [175]. The establishment of a stable genetic transformation system in *Cannabis* deepens our understanding of regulatory mechanisms and developmental biology and is critical in establishing *Cannabis* as a model plant. Genetic improvement to obtain new *Cannabis* varieties with superior traits serves as a valuable resource for the medicinal and health utilization of cannabinoids.

In conclusion, *Cannabis*, an ancient medicinal plant with a longstanding history of global usage over millennia, can make a substantial transformative effect on human health in the future.

## Acknowledgments

This work was supported by the National Natural Science Foundation of China (82204579), the Fundamental Research Funds for the Central Universities (2572022DX06), the Scientific and Technological Innovation Project of China Academy of Chinese Medical Science (CI2021A04113), and Heilongjiang Touyan Innovation Team Program (Tree Genetics and Breeding Innovation Team).

## Author contributions

Z.X., W.S., and S.C. designed and coordinated the review. Z.X. structured, drafted, and revised the manuscript. Y.M, L.K., and M.G. revised the manuscript. S.C., W.C., and X.M. contributed to the critical revision of the manuscript. All authors read and approved the final manuscript.

## Conflict of interest statement

The authors declare that they have no conflict of interest.

## Supplementary Material

Web_Material_uhad150
