# Supplementary material for: Cannabis sativa: origin and history, glandular trichome development, and cannabinoid biosynthesis
Source: Hortic Res. 2023 Jul 26;10(9):uhad150. doi: 10.1093/hr/uhad150 (PMC10485653; doi:10.1093/hr/uhad150)
Supplement: Web_Material_uhad150 [file web_material_uhad150.zip › Supplementary Figure.docx]

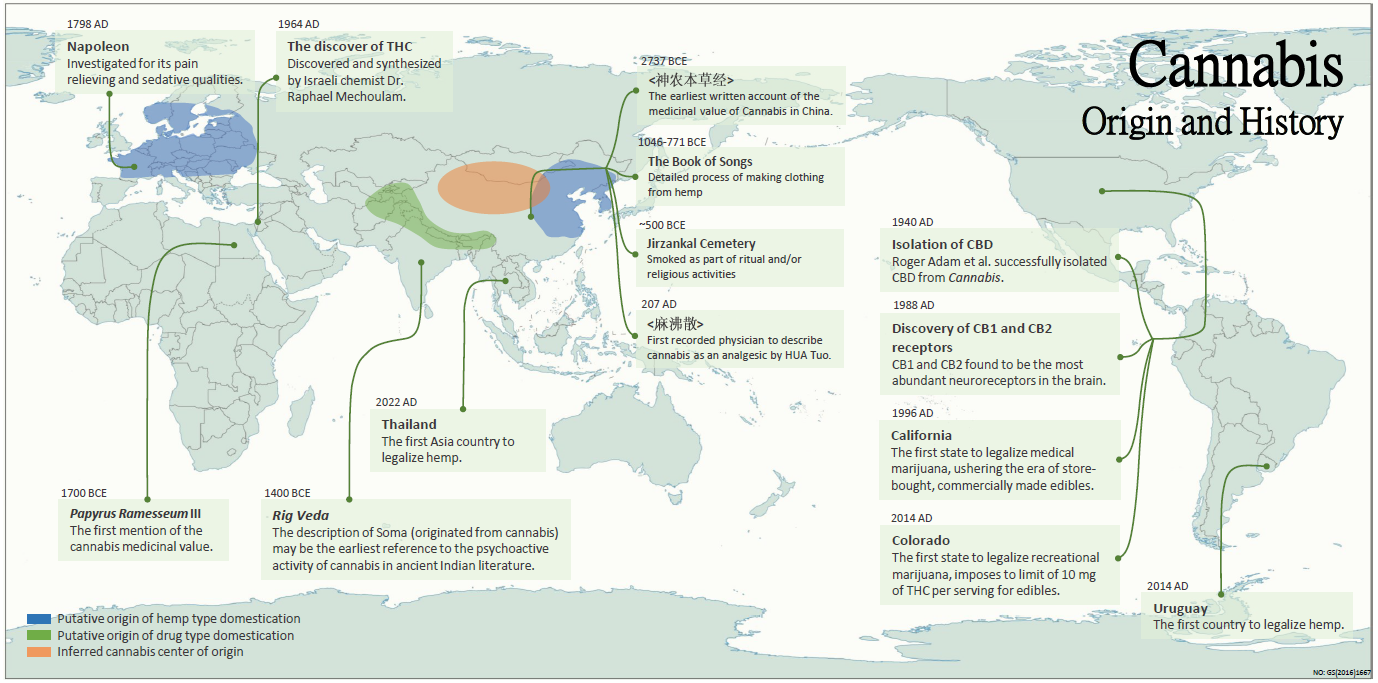


**Fig. S1**: **Important events in the history of *Cannabis sativa* L. and its putative origin.** Major events in the history of *Cannabis* usage and their respective locations have been marked on the map. Orange indicates the inferred center of origin of *Cannabis*. Blue indicates the putative origin of domestication of hemp-type *Cannabis*. Green indicates the putative origin of domestication of drug-type *Cannabis*. Base map was downloaded from http://bzdt.ch.mnr.gov.cn, and the approval number ID is GS(2016)1667.
